# Predictive Biomarkers of Intensive Care Unit and Mechanical Ventilation Duration in Critically-Ill Coronavirus Disease 2019 Patients

**DOI:** 10.3389/fmed.2021.733657

**Published:** 2021-08-12

**Authors:** Sara Taleb, Hadi M. Yassine, Fatiha M. Benslimane, Maria K. Smatti, Sven Schuchardt, Omar Albagha, Asmaa A. Al-Thani, Ali Ait Hssain, Ilhame Diboun, Mohamed A. Elrayess

**Affiliations:** ^1^Division of Genomics and Translational Biomedicine, College of Health and Life Sciences, Hamad Bin Khalifa University, Doha, Qatar; ^2^Biomedical Research Center (BRC), Qatar University, Doha, Qatar; ^3^Department of Bio- and Environmental Analytics, Fraunhofer Institute for Toxicology and Experimental Medicine (ITEM), Hannover, Germany; ^4^Medical Intensive Care Unit, Hamad Medical Corporation, Doha, Qatar

**Keywords:** COVID-19, metabolomics, biomarkers, ICU outcome, ICU management

## Abstract

**Introduction:** Detection of early metabolic changes in critically-ill coronavirus disease 2019 (COVID-19) patients under invasive mechanical ventilation (IMV) at the intensive care unit (ICU) could predict recovery patterns and help in disease management.

**Methods:** Targeted metabolomics of serum samples from 39 COVID-19 patients under IMV in ICU was performed within 48 h of intubation and a week later. A generalized linear model (GLM) was used to identify, at both time points, metabolites and clinical traits that predict the length of stay (LOS) at ICU (short ≤ 14 days/long >14 days) as well as the duration under IMV. All models were initially trained on a set of randomly selected individuals and validated on the remaining individuals in the cohort. Further validation in recently published metabolomics data of COVID-19 severity was performed.

**Results:** A model based on hypoxanthine and betaine measured at first time point was best at predicting whether a patient is likely to experience a short or long stay at ICU [area under curve (AUC) = 0.92]. A further model based on kynurenine, 3-methylhistidine, ornithine, p-cresol sulfate, and C24.0 sphingomyelin, measured 1 week later, accurately predicted the duration of IMV (Pearson correlation = 0.94). Both predictive models outperformed Acute Physiology and Chronic Health Evaluation II (APACHE II) scores and differentiated COVID-19 severity in published data.

**Conclusion:** This study has identified specific metabolites that can predict in advance LOS and IMV, which could help in the management of COVID-19 cases at ICU.

## Introduction

The severe acute respiratory syndrome coronavirus 2 (SARS-CoV-2) remains a major threat worldwide, causing the coronavirus disease 2019 (COVID-19) pandemic that has endangered the lives of millions around the globe. One-fifth of COVID-19 patients exhibits respiratory distress that necessitates instant oxygen therapy or hospital interventions such as invasive mechanical ventilation (IMV) (1, 2). Among the critically-ill patients admitted to the intensive care unit (ICU), one-third of patients dies (3). At times of crises, intensivists are often inclined to predict the duration of IMV for a better utilization of ICU resources. However, the accuracy of early clinical prediction of IMV duration remains limited, especially in patients who will require longer IMV (4). In parallel, markers that can predict patients' evolution at ICU may also be legitimate targets for intervention to improve the patient clinical profile at ICU.

Previous metabolomic studies have compared the metabolic profile of healthy individuals vs. groups of COVID-19 patients with varying degrees of severity (5, 6). However, since the measurements were taken after the phenotypes were acquired, these models could hardly be considered predictive. In this study, we hypothesized that SARS-CoV-2 triggers specific metabolic alterations that can be detected in the sera of intubated patients admitted to ICU and potentially used to differentiate, early on intubation, patients who are more likely to recover from those who would sustain an extended stay at ICU. To test this hypothesis, targeted metabolic profiling was performed to analyze the sera of critically-ill COVID-19 patients in ICU within the first 48 h of intubation and 1 week later. These patients represent the real burden on the health system and are liable to some of the worst possible outcomes of the disease, which renders early prediction of their evolution at ICU of tremendous clinical value.

## Methods

### Study Design

**Figure 1:**
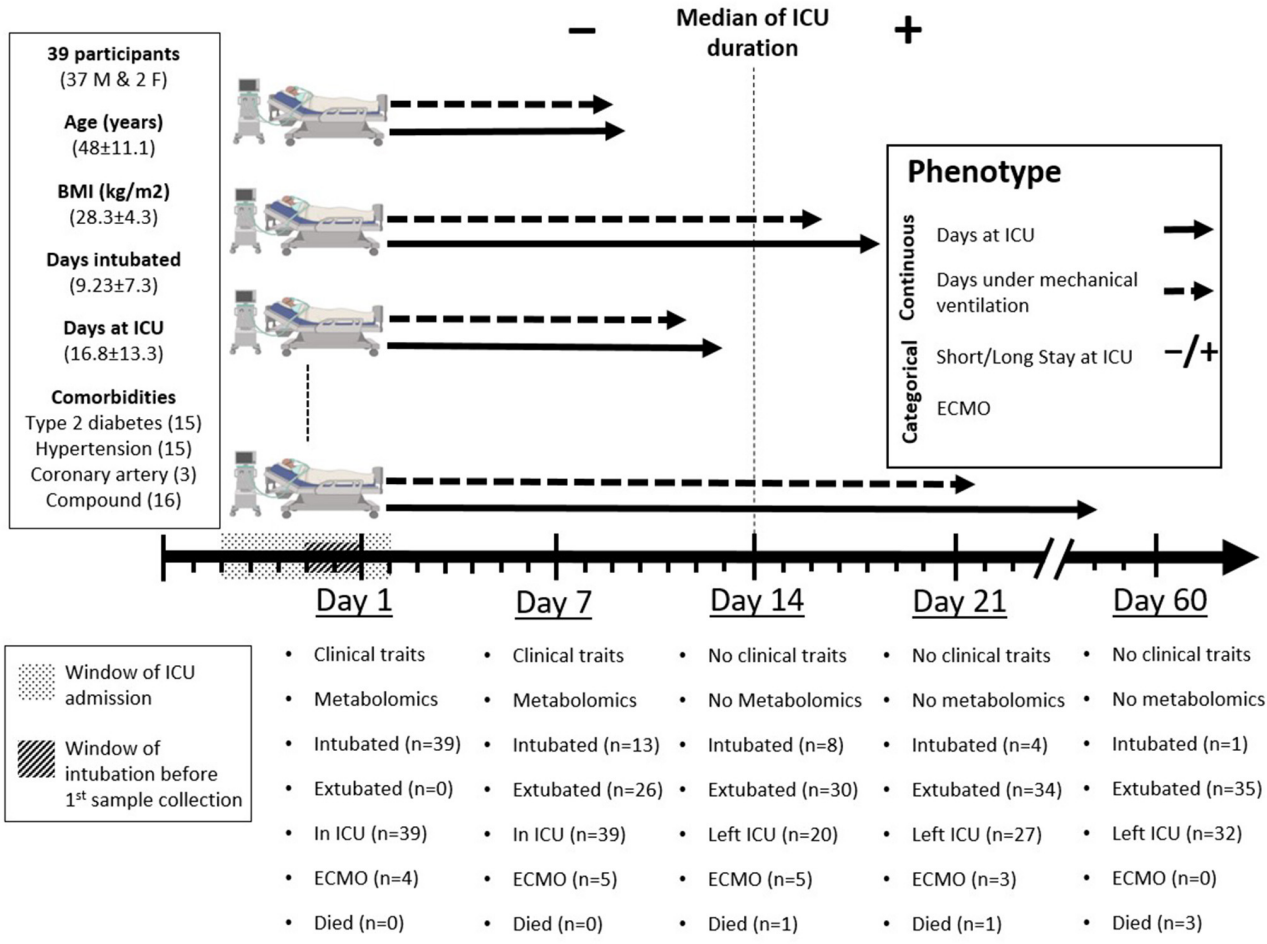
Study design. Day one represents the day of inclusion and first sample collection when all participants were already under mechanical ventilation. Patients' intubation started 2 days before to 4 days after ICU admission (window of intubation). Blood samples were collected from intensive care unit (ICU) patients 1 day before ICU admission to 5 days after ICU admission (window of ICU admission), then 7 days later. Clinical and metabolic profiles were measured at day one and day seven and were correlated with four phenotypes: two continuous (days at ICU and days under mechanical ventilation) and two categorical [short (≤ 14 days) or long (>14 days) stay at ICU and progression to ECMO]. Clinical outcomes were recorded at days one, seven, fourteen, twenty-one, and sixty. Participants' data for age, body mass index (BMI), days under mechanical ventilation, and days at ICU are presented as mean ± standard deviation (SD).

This is a cross-sectional study containing 39 critically-ill COVID-19 patients admitted to ICU at Hamad Medical Corporation (HMC), the main health care provider in Qatar. Protocols were approved by Institutional Review Boards (IRBs) of HMC (MRC-05-007) and Qatar University (1289-EA/20). All methods were performed in accordance with the relevant guidelines and regulations. Informed consents were obtained from all subjects or legal guardian. Demographics, anthropometrics, and medical history data were collected including age, ethnicity, vital signs, body mass index (BMI), comorbidities, complete blood count (CBC), and kidney and liver function. Throughout the article, we refer to these phenotypic measures as clinical traits. The ICU prognostic Acute Physiology and Chronic Health Evaluation II (APACHE II) scoring system was adopted as a predictive measure of death and a correlate of disease severity in critical patients (7, 8). Patients' intubation started from 2 days before ICU admission to 4 days after ICU admission. Blood samples were collected from ICU patients within 48 h of intubation and 7 days later. Time of first sample collection is referred to as day one; similarly, the time of second sample collection is referred to as day seven. Patients were followed up to 60 days after recruitment, and information on days at ICU, days under IMV, progression to extracorporeal membrane oxygenation (ECMO), and deaths was recorded (Figure 1).

### Metabolomics

The targeted metabolomics approach allows for the simultaneous quantification of up to 630 metabolites from 26 compound classes (1 alkaloid, 1 amine oxide, 20 amino acids, 30 amino acid related, 14 bile acids, 9 biogenic amines, 1 carbohydrates and related, 7 carboxylic acids, 1 cresol, 12 fatty acids, 4 hormones, 4 indoles and derivatives, 2 nucleobases and related, 1 vitamin and cofactor, 40 acylcarnitines, 76 phosphatidylcholines, 14 lysophosphatidylcholines, 15 sphingomyelins, 28 ceramides, 8 dihydroceramides, 19 hexosylceramides, 9 dihexosylceramides, 6 trihexosylceramides, 22 cholesteryl esters, 44 diglycerides, and 242 triglycerides) using a combination of liquid chromatography and mass spectrometry. Briefly, a 96-well-based sample preparation device was used to quantitatively analyze the metabolite profile in the serum samples (<50 μl). This device consists of inserts that have been spotted with internal standards, and a predefined sample amount was added to the inserts. Next, a phenylisothiocyanate solution was added to derivatize some of the analytes (e.g., amino acids), and after the derivatization was completed, the target analytes were extracted with an organic solvent, followed by a dilution step.

The obtained extracts were then analyzed by flow injection analysis–tandem mass spectrometry (FIA–MS/MS) using a SCIEX 5500 QTRAP™ mass spectrometer (SCIEX, Darmstadt, Germany) for lipids and liquid chromatography–tandem mass spectrometry (LC–MS/MS) using Agilent 1290 Infinity II liquid chromatography (Agilent, Santa Clara, CA, USA) coupled with a SCIEX 5500 QTRAP™ mass spectrometer (SCIEX, Darmstadt, Germany) for small molecules using multiple reaction monitoring (MRM) to detect the analytes. Data was quantified using SCIEX Analyst® software and imported into Biocrates MetIDQ™ software for calculating analyte concentrations, data assessment, and compilation.

After measurement of 10% of samples, quality control of data was performed to check for variability and batch effects, e.g., based on site or for hexose (indication for degradation of metabolites). The measurement range was defined upfront and instrument parameter was checked.

#### Raw Data Processing

After normalization and pre-processing of the data, MetIDQ™ software (Biocrates) was used for peak integration and calculation of metabolite concentrations. If the measurements were outside the measurable range, values were imputed as follows: concentrations below the detection limit (LOD) was set to half of the lowest measured concentrations. Concentrations below the limit of quantification (LOQ) were set to half of the LOQ. In addition, concentration higher than the highest calibration standard concentration was set to the highest standard concentrations. Concentration of each metabolite was given in micromolars. Raw metabolomics data is publically available (https://doi.org/10.6084/m9.figshare.14954907.v1).

### Statistical Analysis

Clinical traits analysis was carried out using IBM SPSS version 25. Variables with skewed distributions were log-transformed to ensure normality (9). Comparisons were performed with *t*-test, Wilcoxon–Mann–Whitney, and one-way ANOVA as appropriate. Significance was defined as *p* ≤ 0.05. Non-parametric tests were used for comparing ordinal or non-normal variables. Metabolomics data analysis was performed using SIMCA 16.0.2 software (Umetrics, Umea, Sweden) and R version 4.0.2. Data were log-transformed and scaled.

#### Phenotype Definition

**Figure 2:**
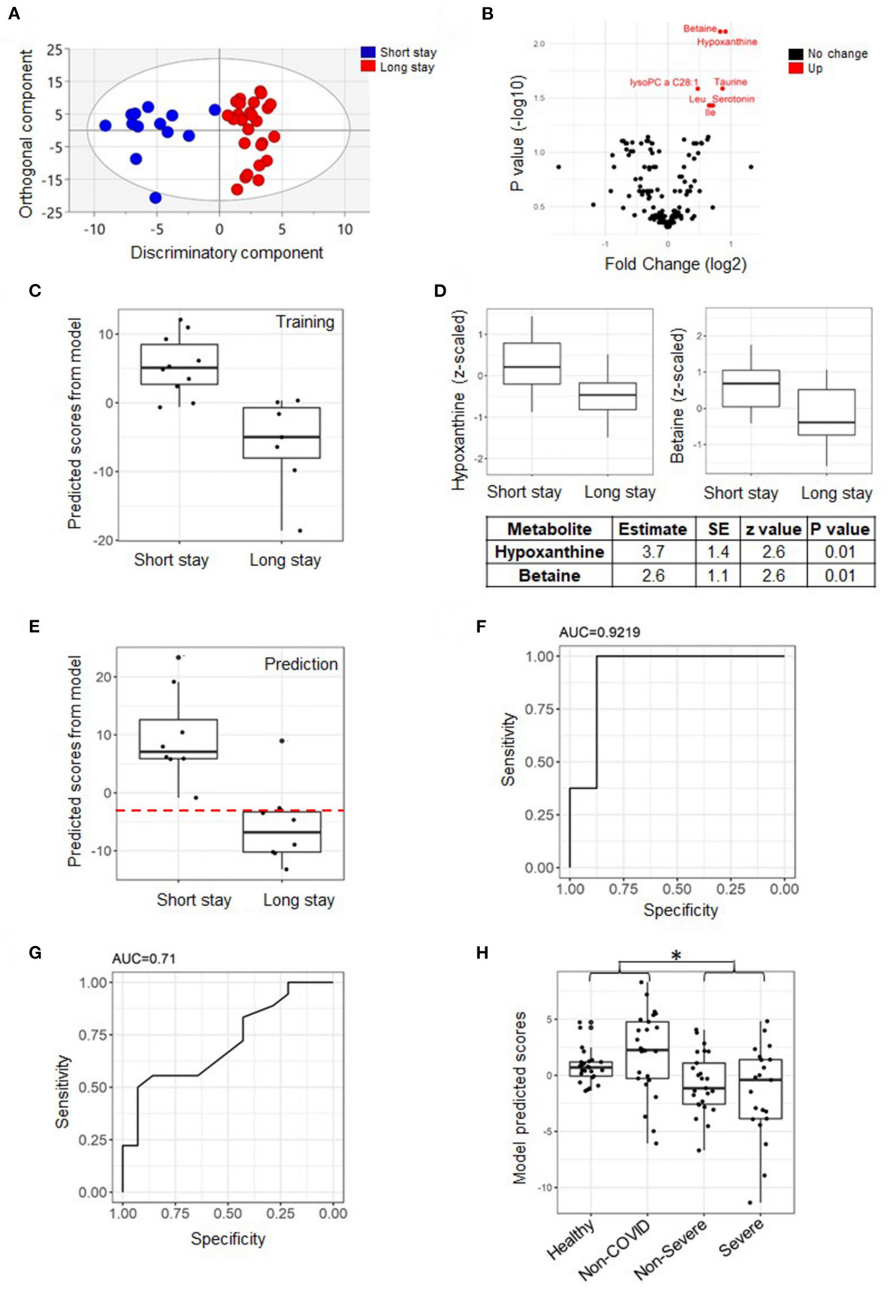
Predictive model of length of stay (LOS) categorized into short/long based on measurement from day one. An orthogonal partial least square discriminant analysis (OPLS-DA) score plot from the whole cohort showing the class-discriminatory component (*x*-axis) vs. the orthogonal confounding component (*y*-axis) for long vs. short LOS groups, the discriminatory component explaining up to 86% of the variation in the Y phenotype variable **(A)**. A volcano plot showing significantly associated metabolites (log fold change >0.06 and adjusted *p* ≤ 0.05) differentiating long from short LOS groups from the linear model based on the training set **(B)**. A predictive model of LOS based on the training set showing perfect separation of patients with short vs. long ICU stay from the same set (*n* = 17) **(C)**. The model featured two explanatory metabolites: hypoxanthine and betaine with independent effects **(D)**. Validation of the model using the prediction set (*n* = 16) and assuming a hypothetical separation line (dashed line in red), the model only misclassified one ICU long-stay patient **(E)**. The area under curve (AUC) value from receiver operating characteristic (ROC) curve analysis was 0.92 **(F)**. Although the Acute Physiology and Chronic Health Evaluation II (APACHE II) score is significantly higher at day one in patients that remain at ICU for longer than 14 days (*p* = 0.01, Table 1), in terms of discriminatory power, it is inferior to our model (AUC = 0.71, *n* = 39) **(G)**. Testing the model on published metabolomics data (28 healthy subjects, 25 non-COVID-19 patients, 25 non-severe COVID-19 patients, and 28 severe COVID-19 patients) revealed that the predicted scores from COVID-19 patients are lower than those from controls and similar to the lower predicted scores by ICU long-stay patients when compared to short stay (*p < 0.001) **(H)**. Data points were slightly scattered across the *x*-axis for ease of visualization in all boxplots.

**Figure 3:**
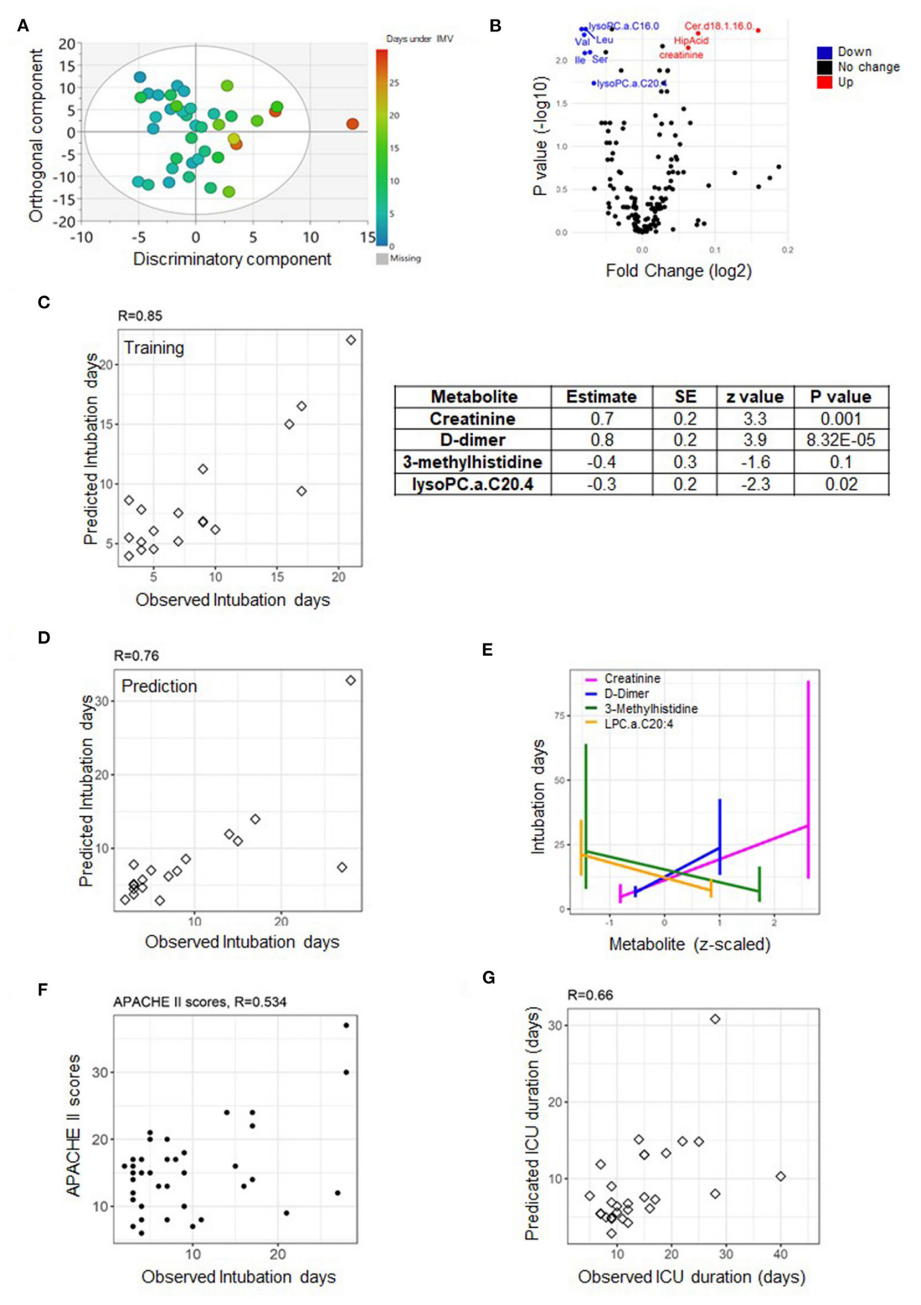
Predictive model of duration of invasive mechanical ventilation (IMV) based on measurements from day one. An OPLS score plot showing the class-discriminatory component (*x*-axis) vs. orthogonal component (*y*-axis) for duration of IMV, the discriminatory component explaining up to 86% of the variation in the Y phenotype variable **(A)**. A volcano plot showing top associated metabolites (log fold change >0.06, adjusted *p* ≤ 0.05) with duration of IMV from the linear model based on the training set **(B)**. The predictive model was trained on metabolites and clinical traits measured from the training set (*n* = 17) on day one **(C)**, then validated on the prediction set (*n* = 16) **(D)**. The model comprised of three metabolites and one clinical trait **(E)** that together showed a better predictive power compared to APACHE II score **(F)**. Using the model to predict the highly correlated number of days at ICU produced a correlation level of 0.66 with their observed counterparts **(G)**.

**Figure 4:**
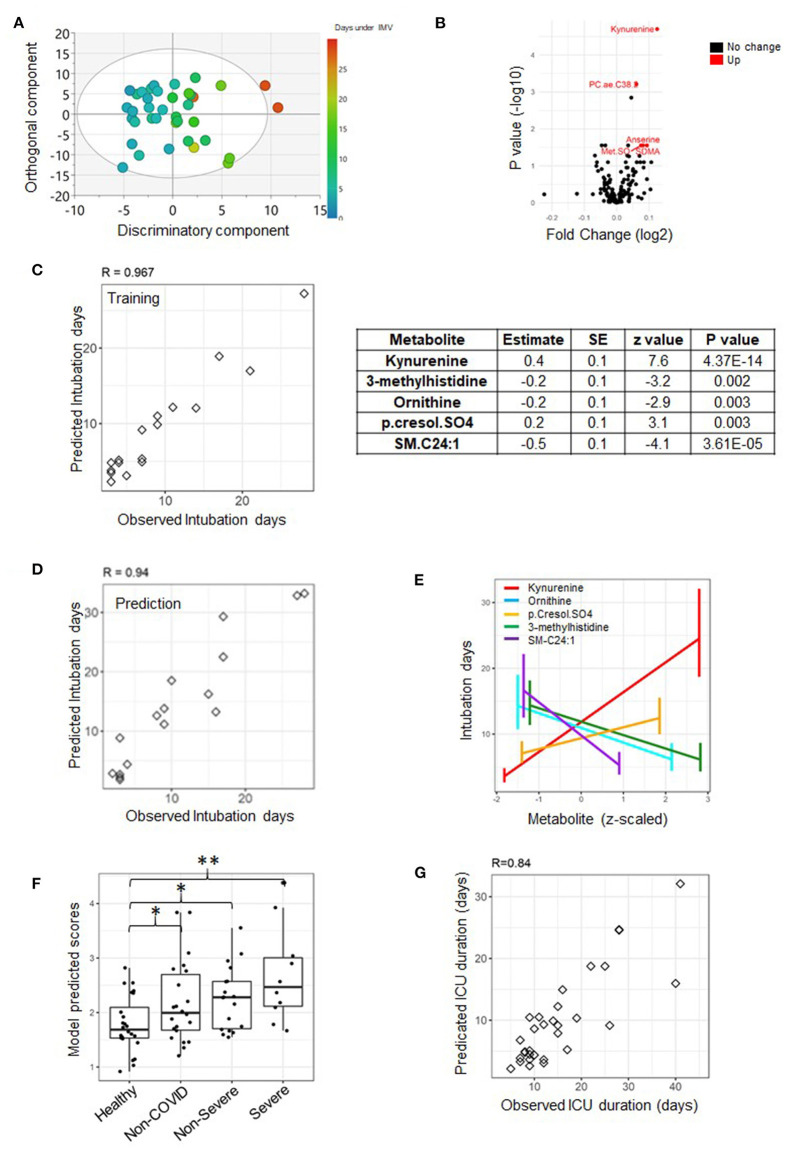
A predictive model of duration of invasive mechanical ventilation (IMV) based on measurements from day seven. An OPLS score plot showing the class-discriminatory component (*x*-axis) vs. orthogonal component (*y*-axis) for duration of IMV, the discriminatory component explaining up to 94% of the variation in the Y phenotype variable **(A)**. A volcano plot showing top associated metabolites (log fold change >0.06, adjusted *p* ≤ 0.05) with duration of IMV from the linear model based on the training set **(B)**. An analysis of the training set (*n* = 17) revealed that the best predictive model only featured metabolites and none of the clinical traits **(C)**, and the model was validated on the prediction set (*n* = 16) **(D)**. The model comprised of five predictive metabolites that either increased or decreased at day seven with longer intubation days **(E)**. When tested on published metabolomics data from non-ICU patients, it could reveal the extent of severity (*p < 0.05, **p < 0.001) **(E)**. Data points were slightly scattered across the *x*-axis for ease of visualization in the boxplot in **(F)**. Using the model to predict the highly correlated number of days at ICU produced a correlation level of 0.84 with their observed counterparts **(G)**, superior to that based on day one.

The specific ICU-related outcomes of interest to this study were the duration of IMV in days and the length of stay (LOS) at ICU given as short/long for patients who spent shorter or longer than 14 days at ICU, respectively. Also, the ECMO status distinguishes patients who required assisted oxygenation via ECMO from those who did not. It is important to note that with respect to LOS, the 14 days cut-off was based on the median of the ICU duration across the cohort (Figure 1). Orthogonal partial least square (OPLS) and its counterpart (OPLS-DA, DA standing for discriminant analysis) from SIMCA were used for QC to examine the separation of samples according to the continuous “duration of IMV” and categorical “LOS” phenotypes, respectively. This was based on metabolomics data measured on both time points, days 1 and 7, separately (Figures 2A, 3A, 4A).

**Table 1:** Clinical features of critically-ill COVID-19 patients.

**Clinical traits**	**A: Overall differences in days 1 and 7**			**B: Based on day 1 measurements**			**C: Based on day 7 measurements**		
	**Day 1 (*n* = 39)**	**Day 7 (*n* = 39)**	***p* value**	**Long stay (>14 days) (*n* = 19)**	**Short stay (<14 days) (*n* = 20)**	***p* value**	**Long stay (>14 days) (*n* = 19)**	**Short stay (<14 days) (*n* = 20)**	***p* value**
Age (years)	48 (11.1)			50 (11.1)	45.6 (11.1)	0.23			
BMI (kg/m^2^)	28.3 (4.3)			29.3 (5.3)	26.9 (2.3)	0.22			
SpO_2_ %	96.8 (1.9)	96.4 (1.9)	0.554	98 (2)	96.3 (2)	0.2	96.2 (2)	96.7 (1.9)	0.51
Art pH	7.4 (0.1)	7.5 (0.1)	<0.001	7.4 (0.1)	7.3 (0.1)	0.57	7.4 (0.1)	7.5 (0.1)	0.08
PaO_2_ (mmHg)	77.9 (19.4)	88.5 (45.7)	0.446	71 (22)	80.2 (22)	0.5	94.3 (20.8)	79.3 (22.1)	0.45
PaCO_2_ (mmHg)	43 (5.5)	40 (8.1)	0.247	41.7 (5.3)	43.4 (5.3)	0.65	41.6 (8)	36.7 (8.2)	0.15
Lactate (mmol/l)	1.1 (0.3)	1.6 (0.6)	0.023	0.9 (0.3)	1.2 (0.3)	0.16	1.7 (0.4)	1.3 (0.4)	0.12
Creatinine (umol/l)	108.5 (60.5)	115.4 (95)	0.811	161 (26.2)	92.8 (26.2)	0.09	144.8 (39.7)	84.1 (28.5)	0.06
T. bilirubin (umol/l)	11 (3.6)	31.4 (56.7)	0.223	14.3 (2.5)	9.9 (2.5)	0.06	49.3 (8.3)	16.5 (8.5)	0.1
T. protein (g/l)	66.1 (5.3)	63.5 (9.5)	0.386	63.5 (5.8)	66.7 (5.8)	0.48	63.3 (7.1)	63.3 (7.3)	1
Triglycerides (mmol/l)	1.7 (0.5)	3.7 (1.7)	0.002	1.5 (0.5)	1.8 (0.5)	0.6	4.2 (0.6)	2.9 (0.6)	0.05
ALT (U/l)	45.7 (34.5)	119.9 (83.3)	0.005	30.3 (38.6)	50.8 (38.6)	0.4	126.6 (66.4)	113.6 (66.4)	0.66
AST (U/l)	47.5 (28.5)	69 (62.6)	0.261	44 (33.1)	48.7 (33.1)	0.82	68.5 (78.6)	69.5 (78.6)	0.96
ALP (U/l)	66.4 (20.9)	97.8 (76.3)	0.153	61 (23.2)	68 (23.2)	0.63	118.7 (41.9)	76.9 (40.5)	0.13
Albumin (g/l)	25.5 (3.1)	28.6 (4.8)	0.035	26.3 (3.4)	25.3 (3.4)	0.63	27.9 (4.5)	29.2 (4.6)	0.44
Glutamine (mmol/l)	11.3 (4.6)	10.4 (4.4)	0.621	9.3 (5.3)	12.1 (5.3)	0.42	11.8 (3.8)	9.3 (3.8)	0.11
Cholesterol (mmol/l)	102.1 (4.2)	104.6 (6.8)	0.203	102.7 (4.3)	101.9 (4.3)	0.79	104.5 (6)	104.5 (6.1)	0.99
Bicarbonate (mmol/l)	21.9 (2.9)	25.5 (4.4)	0.012	22 (2.5)	21.9 (2.5)	0.96	26.5 (3.6)	24.5 (3.7)	0.18
Sodium (mmol/l)	138.2 (4.7)	142.3 (7.2)	0.064	137.3 (3.3)	138.5 (3.3)	0.72	144 (7.1)	140.4 (7)	0.13
Potassium (mmol/l)	4.3 (0.7)	4.3 (0.5)	0.922	4.1 (0.7)	4.4 (0.7)	0.58	4.5 (0.4)	4.2 (0.5)	0.11
Magnesium (mmol/l)	1.1 (0.2)	1 (0.1)	0.281	0.9 (0.2)	1.2 (0.2)	0.22	1.1 (0.1)	1 (0.1)	0.1
Phosphate (mmol/l)	1 (0.2)	1.3 (0.3)	0.022	0.9 (0.2)	1 (0.2)	0.72	1.3 (0.2)	1.2 (0.2)	0.59
Urea (mmol/l)	7 (3.4)	15 (9.6)	0.075	10 (3.9)	7.8 (3.8)	0.13	18.7 (4.8)	11.2 (4.5)	0.02
Calcium (mmol/l)	1.9 (0.5)	2.7 (3.6)	0.487	1.7 (0.5)	2 (0.5)	0.5	3.4 (0.3)	2.1 (0.3)	0.33
Fibrinogen (g/l)	6.5 (1.9)	2.9 (1.1)	<0.001	5.5 (2.1)	6.9 (2.1)	0.41	2.7 (1.3)	3.2 (1.3)	0.27
D-dimer (mg/l)	4.8 (7.4)	5.4 (5.5)	0.8	11 (4.6)	3 (4.6)	0.19	6.5 (4.6)	4 (4.8)	0.26
Ferritin (μg/l)	1,346.1 (1,039.8)	1,255.9 (823.7)	0.766	680 (1,120.5)	1,568.1 (1,120.5)	0.21	1,227.1 (624.9)	1,286.7 (624.9)	0.84
RBC (×10^6^/μl)	4.7 (1)	4.4 (1.1)	0.291	4.1 (1)	4.9 (1)	0.35	3.8 (1)	5 (1)	<0.001
Hemoglobin (g/dl)	12.4 (1.7)	12 (2.6)	0.679	11.9 (1.7)	12.5 (1.7)	0.62	10.6 (2.3)	13.5 (2.2)	<0.001
Hematocrit %	36.7 (5.4)	37.2 (8.1)	0.868	35.4 (5.1)	37.3 (5.1)	0.63	33.4 (7)	41.4 (6.7)	<0.001
MCHC (g/dl)	32.6 (1.7)	32.3 (1.4)	0.604	32.7 (64.3)	46.7 (65.8)	0.42	32.1 (1.3)	32.5 (1.3)	0.34
MCH (pg)	26.4 (3.6)	28.1 (2.5)	0.106	28.4 (2.6)	26.8 (2.6)	0.12	28.6 (1.8)	27.5 (1.7)	0.16
MCV (fL)	80.6 (8.7)	86.9 (7.7)	0.032	79.3 (9.8)	81 (9.8)	0.82	89.1 (4.6)	84.5 (4.2)	0.08
MPV (fL)	10.6 (0.9)	11 (1.2)	0.421	10.2 (1)	10.8 (1)	0.48	11.4 (1.1)	10.7 (1.1)	0.07
PDW (fL)	13.3 (2.8)	13.8 (2.9)	0.648	11.8 (3)	13.7 (3)	0.42	14.8 (2.5)	13.2 (2.6)	0.14
RDW-CV %	14.5 (1.7)	14 (1.7)	0.421	14.4 (1.7)	14.5 (1.7)	0.92	14.3 (1.7)	13.5 (1.4)	0.14
WBC (×10^3^/μl)	10.3 (3.5)	15.9 (6.2)	0.005	11.1 (2.9)	10 (2.9)	0.64	17.2 (4.6)	14.7 (4.7)	0.25
ANC (×10^3^/μl)	9.5 (3.4)	13.5 (6.6)	0.101	10.7 (3.3)	9 (3.3)	0.58	15.8 (5.1)	11.3 (5.2)	0.05
Neutrophils (%)	87.6 (4.6)	83.7 (11.4)	0.325	86 (3.9)	88.4 (3.9)	0.5	88.9 (13.2)	77.9 (13.1)	<0.001
Basophils (×10^3^/μl)	0 (0)	0.1 (0.1)	0.495	0 (0)	0 (0)	0.52	0.1 (0)	0 (0)	0.22
Basophils (%)	0.3 (0.2)	0.3 (0.2)	0.916	0.2 (0.2)	0.3 (0.2)	0.3	0.3 (0.2)	0.3 (0.2)	0.65
Eosinophils (×10^3^/μl)	0 (0)	0.1 (0.2)	0.064	0.01 (0.11)	0.03 (0.12)	0.6	0.1 (0.2)	0.2 (0.2)	0.03
Eosinophils (%)	0.1 (0.1)	0.9 (1.2)	0.06	0 (0.2)	0.1 (0.2)	0.6	0.3 (1.4)	1.3 (1.4)	0.02
Lymphocytes (×10^3^/μl)	0.9 (0.3)	1.7 (3.2)	0.421	0.9 (0.3)	0.9 (0.3)	0.91	1.9 (1)	1.7 (1)	0.83
Lymphocytes (%)	8.4 (3.3)	9.1 (7.7)	0.791	8.8 (2.1)	8.2 (2.1)	0.87	4.9 (8.6)	13.4 (8.5)	<0.001
Monocytes (×10^3^/μl)	0.4 (0.2)	0.8 (0.4)	0.022	0.5 (0.2)	0.4 (0.2)	0.59	0.7 (0.4)	0.9 (0.4)	0.14
Monocytes (%)	4 (1.9)	5.7 (3.3)	0.173	4.2 (2.1)	3.9 (2.1)	0.88	4.3 (3.9)	7.2 (3.8)	0.01
Platelets (×10^3^/μl)	312.6 (102.1)	343.5 (169)	0.572	297.5 (108.7)	316 (108.7)	0.83	297.2 (175.4)	385 (175.1)	0.16
APTT (s)	36.4 (18.3)	28.4 (9.9)	0.099	31.8 (20.9)	37.7 (20.9)	0.71	30.1 (4.3)	26.7 (4.3)	0.36
PTT (s)	18.5 (11.1)	12.8 (1.5)	0.008	13 (12.3)	20.4 (12.3)	0.45	12.8 (0.9)	12.8 (0.9)	0.92
CRP (mg/l)	281.7 (164.1)	29.4 (45.4)	<0.001	159 (107.5)	208.5 (106.9)	0.16	22.7 (72.7)	39.5 (72.7)	0.5
APACHE II score	15.1 (6.4)	N/A	N/A	17.8 (4.4)	12.7 (4.4)	0.01	

*The table summarizes differences in clinical features between (A) all patients at day one and day seven, (B) patients who remained at ICU for more than 14 days (long stay) from those who left before 14 days (short stay) based on measurement on day one or (C) measurement on day seven. Abbreviations: BMI, body mass index; ALP, alkaline phosphatase; ALT, alanine transaminase; AST, aspartate aminotransferase; SpO_2_ %, oxygen saturation; Art pH, arterial pH; PaO_2_, partial pressure of oxygen; PaCO_2_, partial pressure of carbon dioxide; RBC, red blood cells; MCHC, mean corpuscular hemoglobin concentration; MCH, mean corpuscular hemoglobin; MPV, mean platelet volume; PDW, platelet distribution width; RDW, red cell distribution width; WBC, white blood cells; ANC, absolute neutrophil count, aPTT, activated partial thromboplastin time; PTT, partial thromboplastin time; CRP, C-reactive protein; APACHE II, a severity-of-disease classification system. Data are presented as mean (SD). Differences between groups were tested by independent sample t-test (normally distributed variables) or Mann–Whitney U (variables with skewed distribution) test. A p-value significance level of 0.05 was used*.

#### Variable Selection

The primary goal of this study was to build statistical models that can predict our ICU phenotypes of interest. To this end, the cohort was randomly split into a training and a validation set. The fact that the number of metabolites and clinical traits far exceeded the number of individuals in our cohort complicates the statistics of fitting a predictive model. Therefore, subsets of markers significantly associated with the phenotypes of interest needed to be determined *a priori*. These would serve as seed variables on which to train the predictive models for the target phenotypes. The identification of such subset was performed on the training set in two steps: First, each trait was associated with the phenotype of interest in a general linear model. With metabolites, the model also incorporated principal components (PC) PC1 and 2, from principal component analysis (PCA), BMI, and age as confounders:

$$\begin{array}{l} Y_{\text{metabolite}}\sim \text{age}+\text{BMI}+\text{PC}1+\text{PC}2+\text{ phenotype }{\text{Y}}_{\text{trait}}\sim phenotype \end{array}$$

Second, we used the elastic net-regularized extension of the generalized linear model, implemented in the R package GLMNET, to regress the phenotypes of interest on the measured metabolites as follows:

$$\begin{array}{l} {\text{Y}}_{\text{phenotype}}\sim \text{metabolit}{\text{e}}_{1}+metabolite_{2}+\ldots +metabolite_{\text{n}} \end{array}$$

Since the GLMNET accepts no missing values, we therefore removed samples where the significant metabolites from the linear model (step 1) were not measured, then omitted metabolites with missing values in the remaining samples. The advantage of the GLMNET analysis is its ability to deal with a large number of explanatory variables at once whereby the association of each metabolite with the phenotype is assessed while accounting for the effect of the remaining metabolites. However, the GLMNET framework is mostly mathematical and offers little statistical properties in terms of model fit, which justifies the following additional analysis step.

#### Predictive Model Formulation

We used the generalized linear model based on the binomial distribution for the categorical phenotypes (ECMO and ICU stay) as oppose to the Poisson distribution for modeling the number of days under IMV. Each model was fit on the training set and featured all metabolites promoted by the GLMNET as well as traits found significant from the initial linear model. The model was then refined in a stepwise procedure, omitting a variable each time and reassessing the fit until the best explanatory subset of variables was found. The evaluation of the model was based on the Pearson correlation between observed and predicted days under IMV for the remaining samples in the cohort or the prediction set. As for the categorical phenotypes, receiver operating characteristic (ROC) curve analysis, sensitivity, and specificity measures at median predicted value were used. Owing to explanatory variable missing values, the predictive models for duration under IMV and LOS were based on a subset of the cohort with *n* = 33 (17 training/16 prediction) out of a total of 39. It is important to note that for all phenotypes, a model was constructed at each time point separately.

#### Model Additional Validation on Published Data

We used published metabolomics data (5) measured from a cohort of *n* = 106 individuals comprising of 28 healthy subjects, 25 non-COVID-19 patients (negative for the SARS-CoV-2 nucleic acid test) with similar clinical characteristics as COVID-19 patients, 25 non-severe COVID-19 patients, and 28 severe COVID-19 patients. The published data in question was further processed by log transformation and *z*-scaling.

## Results

### General Characteristics of Participants

Thirty-nine mid-age (48 ± 11.1 years) critically-ill patients were recruited among patients admitted to the ICU at HMC. Among recruited patients, 15 (38.5%) patients had type 2 diabetes, 15 (38.5%) patients had hypertension, and three (7.7%) had coronary artery disease (CAD). All patients were under IMV on day one of sample collection. Patients spent on average 16.8 days (SD = 13.3) at ICU with a median of 14, of which 9.2 days (SD = 7.3) were under IMV. At week two (day 7), 26 patients (66.7%) were extubated. At week three (day 14), 30 patients (76.9%) were extubated, 20 (51.3%) left the ICU, and one (2.6%) died (Figure 1). Differences in clinical features of study participants based on their test results on day one and day seven are summarized in Table 1(A). Certain clinical features significantly increased during the 1st week at ICU, including arterial pH (pH art), serum lactate, triglycerides, alanine aminotransferase (ALT), albumin, bicarbonate, phosphorus, mean corpuscular volume (MCV), white blood cells (WBC), percentage of monocytes, and C-reactive protein (CRP), whereas both fibrinogen and partial thromboplastin time (PTT) were significantly reduced during the 1st week at ICU. Differences in clinical features between patients who remained in the ICU (long stay) and those discharged prior to day 14 (short stay) based on measurement on day one are shown in Table 1(B). Among the clinical features measured on day one, only APACHE II score was significantly lower in patients who subsequently left ICU before day 14. Among the clinical traits measured at day 7, the data suggest that patients who left ICU earlier than day 14 had significantly higher red blood cell count (RBC), hemoglobin, percentage hematocrit, and the number of lymphocytes and monocytes, but lower triglycerides, urea, absolute neutrophil count (ANC), and percentage of neutrophils and eosinophils [Table 1(C)].

### A Multivariate Predictor of Categorized LOS at ICU

It is of clinical interest to predict the likely pattern of ICU duration for critically-ill patients on admission. An initial linear model based on measurements taken within 48 h of admission to ICU (day 1) highlighted a strong signature by hypoxanthine and betaine (Figure 2B). A further analysis of traits and the remaining metabolites with no missing values using the GLMNET statistics followed by model refinement (refer to *Methods* section) identified hypoxanthine and betaine as the sole best predictors based on the training set (*n* = 17) (Figure 2C). Both metabolites were lower on day one of sample collection among patients likely to remain at ICU for longer than a 2-week period, and their effects were independent of each other (Figure 2D). When tested on the prediction set (*n* = 16) (Figure 2E), the model scored an area under curve (AUC) value of 0.92 (95% CI: 0.76–1) as well as a sensitivity and specificity values of 0.875 and 0.875, respectively (Figure 2F). Only one out of the eight long-ICU-stay patients was mistakenly assigned to the short-stay category by the model (dashed line, Figure 2E). The hypoxanthine/betaine model appeared to outperform the APACHE II scores that discriminated short from long ICU state with an AUC value equal to 0.71 (also a sensitivity and specificity measure both equal to 0.4) (Figure 2G). The model was then tested on published metabolomics data (5). Although the cohort focused on different categories of COVID-19 patients (mild and severe non-ICU patients), the general trend was the same: The predicted scores for the COVID-19 patients (non-severe and severe combined) were significantly less than the average from controls together with the non-COVID-19 patients (*p* = 0.000895) (Figure 2H). The model, however, did not significantly differentiate the varying COVID-19 severity levels prior to ICU.

We also sought to model the categorized LOS at ICU based on metabolomics measurements on day seven. This model is still predictive since no patient left the ICU before week one. Similar to the previous model, hypoxanthine and betaine were found to be the best explanatory variables of LOS, although the associated AUC value was 0.81 (95% CI: 0.596–1), inferior to that from the previous model (Supplementary Figure 1).

### A Multivariate Predictor of Duration of IMV

The availability of respiratory ventilators at ICU can be a limiting factor at times of pandemic crisis. The linear model suggested significant associations with Cer.d18.1.16.0, C16.0 Lyso.PC, Var, Leu, Ile, Ser, creatinine, and C20.4 LysoPC (absolute log fold change >0.06 and *p* ≤ 0.05) measured on day one of sample collection from patients assigned to the training set (Figure 3B). However, based on the totality of clinical traits and metabolites, the best explanatory subset by the GLMNET analysis (refer to *Methods* section) was found to include one trait: D-dimer (a fibrin degradation product) and metabolites creatinine and lysophosphatidylcholine C20:4 (C20:4 LysoPC) (both identified as significant from the linear model) in addition to 3-methylhistidine (with a borderline effect) (Figure 3C). The Pearson correlation (*R*-value) between the observed and predicted number of days under IMV was 0.85 (*p* = 7.28e-06) and 0.76 (*p* = 0.0006) for the training (*n* = 17) and prediction set (*n* = 16), respectively (Figures 3C,D). A close examination of the identified explanatory variables revealed an increase in the level of D-dimer as oppose to a decrease in creatinine, 3-methylhistidine, and lysophosphatidylcholine C20:4 levels soon after admission to ICU with longer intubation periods (Figure 3E). This is superior to the correlation with the APACHE II scores, found equal to 0.53 (*p* = 0.0006) (Figure 3F). Since the number of days at ICU and under IMV is highly concordant (*R* = 0.7, *p* = 0.0014), we used the same model to predict the former. The correlation level between the observed and predicted days at ICU was 0.66 (*p* = 2.39e-05) (Figure 3G).

A superior predictive model of the length of IMV was obtained from measurements taken on day seven. The model featured five metabolites but no clinical trait. The metabolites were kynurenine (also highlighted by the linear model statistics, Figure 4B), 3-methylhistidine, ornithine, p-cresol sulfate, and C24.1 sphingomyelin (Figure 4C). The observed and predicted intubation days by the model are highly concordant: 0.97 (*p* = 1.39e-10) and 0.94 (*p* = 6.52e-07) for the training and prediction set, respectively (Figures 4C,D). A close examination revealed an increase in the level of kynurenine and p-cresol sulfate with longer intubation days as opposed to a decline in the levels of the rest of the explanatory variables on day seven (Figure 4E). The model is superior to its counterpart from measurements taken on day one; however, it is only truly predictive of intubation times longer than 7 days. Interestingly, the model appears to accurately reproduce the severity levels of disease from the published metabolomics study by Shen et al. (5) (Figure 4F). Taking the severity level of patients (from published data) as an ordinal variable, the association with the model-predicted values was significant (*p* = 0.0004). The predicted scores for the COVID-19 patients (non-severe and severe) were significantly greater than the controls (*p* = 0.028 and *p* = 0.0004, respectively) (Figure 4F). It is important to note that verification of the statistical model from day one using the published dataset was not possible due to the unavailability of D-dimer measurement. A better prediction of days at ICU was achieved based on the current model with a Pearson correlation value between observed and predicted days equal to 0.84 (*p* = 3.51e-07) (Figure 4G).

### A Multivariate Predictor of ECMO

A model for ECMO treatment was obtained on the cohort after removing the missing values (*n* = 36) due to the small number of ECMO-positive cases among our patients. The model was entirely based on day one measurement since four of our five patients required ECMO within a week following admission to ICU (Supplementary Figure 2a). It follows that the model was not validated with a prediction set. When accounting for the effect of clinical traits, arterial pH and counts of WBC were jointly found to be the best predictors. Metabolomics measurement offered little improvement into the model's ability to explain the risk of necessitating ECMO (Supplementary Figure 2b). In other words, the model was entirely based on the two identified clinical traits, a close examination of which revealed that patients likely to require ECMO are those with lowest levels of arterial pH and highest counts of white blood cells early on ICU admission/intubation. The model requires validation with a separate dataset.

## Discussion

One of the most challenging aspects of COVID-19 pandemic is managing critically-ill patients at ICU, especially at times of disease peak due to limited capacity for those who require long care. Therefore, it has become imperative to identify patients who are more likely to recover and their expected duration of recovery for better management of resources at ICU. The emerging novel data revealed changes in clinical traits between the initial phase of intubation (within 48 h of intubation) and a week later (refer to Supplementary Material for an elaboration on the biological significance of these effects). More importantly, the data suggest that in addition to clinical traits, molecular changes at the metabolite level at the early phase of admission to ICU and a week later can be used to prospectively predict various severity ICU outcomes.

Based on both time points (days one and seven), hypoxanthine and betaine were together the best predictors of the likelihood of long from short stay at ICU (LOS). Hypoxanthine, a product of purine degradation that results in uric acid as a final product, was previously shown to be associated with malnutrition at ICU (10). Lower hypoxanthine could also indicate increased xanthine oxidoreductase activity converting hypoxanthine to xanthine. Such increased activity could trigger reactive oxygen species (ROS) production that is believed to play a role in the pathogenesis of COVID-19, causing cell necrosis (11). Betaine, an alpha amino acid, was previously shown to act as an osmolyte and a methyl donor in many pathways, including the methylation of homocysteine to methionine (12). Low levels of betaine are associated with increased risk of metabolic diseases (12–14); therefore, lower levels in patients who maintain LOS at ICU could reflect their underlying metabolic diseases.

Three metabolites and one clinical trait were identified as best predictors of longer intubation days based on day one. These included early-admission elevated creatinine and D-dimer as oppose to reduced 3-methylhistidine and lysoPC.a.C20.4. Creatinine is an indicator of impaired kidney function while D-dimer is an indicator of increased coagulation. Both 3-methylhistidine and lysoPC.a.C20.4 are signaling molecules that modulate inflammation. During muscle protein degradation, 3-methylhistidine is released into the circulation and then excreted in the urine. In healthy adults, the ratio of 3-methylhistidine to creatinine excretion remains constant. Muscle protein turnover has been found to be useful for the diagnosis of frailty and sarcopenia (15). Urinary concentration of 3-methylhistidine is a biomarker for skeletal muscle protein breakdown in humans who have been subject to muscle injury (16). In patients with unfavorable evolution of disease, 3-methylhistidine was higher in the plasma metabolome (17). LysoPC.a.C20.4 is a member of the lysophosphatidylcholines that form an important source of diacylglycerides and structural components of the plasma membrane involved in membrane-mediated cell signaling (18). The ratio of lysophosphatidylcholines to phosphatidylcholines is an indicator of phospholipase A2 activity. Increased serum levels of the lipoprotein-associated enzyme reflect intravascular inflammation and are associated with an elevated risk for cardiovascular disease (19, 20).

A better predictive model of duration under IMV was obtained using five metabolites measured on day seven, although effectively only predictive of intubation days longer than a week. It was found that elevated kynurenine and p.cresol.SO_4_, as opposed to lower levels of 3-methylhistidine, ornithine, and SM.C24.1 on day seven post recruitment, are together the best predictors of longer durations of IMV to come. Kynurenine is an indicator of indoleamine 2,3,-dioxygenase (IDO) activity degrading tryptophan to kynurenine. Kynurenine was shown previously to regulate immunity and inflammation (21). p-cresol-SO_4_ synthesis is an indicator of the degradation of the aromatic amino acid tyrosine to para (p)-cresol sulfate by gut bacteria activity (22). It is a uremic toxin associated with kidney disease (23). The model also indicated 3-methylhistidine that belongs to histidine derivatives, ornithine that plays a role in urea cycle, and SM.C24.1 shown previously to correlate with body fat mass (24). Ornithine is an indicator of arginase activity. Arginase is an enzyme that catalyzes the last step in the urea cycle, converting arginine to ornithine, and its activity serves as an indicator for inflammation and potential predictor of mortality in sickle cell disease (25). Lower ornithine could be an indicator of the conversion of ornithine to proline by ornithine aminotransferase and pyrroline-5-carboxylate reductase and an indirect indicator of the activity of arginase to nitric oxide synthase (NOS). An increased ratio indicates that arginase activity, converting arginine to ornithine, is higher than NOS activity, converting arginine to citrulline. In addition, an increased ratio has been shown to be associated with an elevated risk of metabolic syndrome (26). Lower SM.C24.1 levels could indicate lower sphingomyelin synthesis from ceramides facilitated by sphingomyelin synthase. Lower SM.C24.1 was also associated with reduced body fat mass (24). It was interesting to note how clinical traits were most helpful at the early prognosis of the duration of IMV on admission, while metabolite measurements were alone highly predictive a week later. This could be due to the highly diverse profile of comorbidities among ICU patients and how the individualized on-site care, in time, helps to stabilize their overall clinical state, reflected in part by their metabolite measurements.

Since only five patients needed ECMO in our cohort, it was not possible to divide data into training and prediction sets. Therefore, a logistic model explaining the risk of ECMO was trained on the whole dataset instead (excluding samples with missing values of explanatory variables). The model was based on two clinical traits (arterial PH and counts of white blood cells), but no metabolites as explanatory variables. Reduced arterial pH as opposed to elevated white blood cell count early on intubation time was found to be associated with increased risk of ECMO in the following week. While arterial pH is an indicator of respiratory/metabolic acidosis, the count of white blood cells indicates a hyper immune response, which could indicate an overall worst clinical state.

Our study is limited by a small size of the cohort, and future validation of the emerging models in larger cohorts is warranted, in particular during future waves of COVID-19 outbreak. Future studies focusing on identifying the predictive metabolic biomarkers of disease progression in COVID-19 patients at different stages of disease and in relation to various phenotypes including antibody titers are warranted.

## Conclusion

In summary, while a reliable prediction of the number of days under IMV (and the highly correlated number of days at ICU) was possible based on measurements taken on day seven, it is possible to discriminate patients who are generally about to have a short vs. long stay at ICU early on admission. In general, the identified biomarkers were associated with not only the medical complications to COVID-19, including inflammation, coagulation, and kidney injuries, but also the immune response. The proposed models outperformed the predictive ability of the APACHE II score, which, although typically used as a predictive measure of fatality, is also generally accepted as a measure of disease severity.

## Data Availability Statement

The datasets used and/or analyzed during the current study are available from the corresponding author on reasonable request. Raw metabolomics data is publically available (https://doi.org/10.6084/m9.figshare.14954907.v1).

## Ethics Statement

The studies involving human participants were reviewed and approved by Institutional Review Boards (IRBs) of HMC (MRC-05-007) and Qatar University (1289-EA/20). The patients/participants provided their written informed consent to participate in this study.

## Author Contributions

ST, HY, FB, MS, SS, OA, AA-T, AA, ID, and ME contributed to sample collection, analysis, paper writing and review, and acceptance of the final version. ME was responsible for the integrity of the work as a whole. All authors contributed to the article and approved the submitted version.

## Conflict of Interest

The authors declare that the research was conducted in the absence of any commercial or financial relationships that could be construed as a potential conflict of interest.

## Publisher's Note

All claims expressed in this article are solely those of the authors and do not necessarily represent those of their affiliated organizations, or those of the publisher, the editors and the reviewers. Any product that may be evaluated in this article, or claim that may be made by its manufacturer, is not guaranteed or endorsed by the publisher.

## Supplementary Material

Supplementary Figure 1 A predictive model of length of ICU stay (LOS) based on measurement from day seven. The predictive model trained on the training set (*n* = 17) **(a)** featured the same two explanatory metabolites as its counterpart from day one in Figure 2. The model was validated using a prediction set (*n* = 16) **(c)**, and the AUC value from ROC curve analysis was 0.81 **(d)**. Data points were slightly scattered across the *x*-axis for ease of visualization in all boxplots.

Supplementary Figure 2 A predictive model of ECMO status. The model identified on the whole cohort omitting samples with missing explanatory variable values **(a)** featured two explanatory traits: arterial pH and count of white blood cells (WBC) **(b)**. Only five patients required ECMO in our cohort. Due to this small number, the model was trained on the entire dataset, and no prediction set was available for validation. WBC was log-transformed for normality. Data points were slightly scattered across the *x*-axis for ease of visualization in **(a)**.
