# Supplementary figures and images for: Predictive Biomarkers of Intensive Care Unit and Mechanical Ventilation Duration in Critically-Ill Coronavirus Disease 2019 Patients
Source: Front Med (Lausanne). 2021 Aug 12;8:733657. doi: 10.3389/fmed.2021.733657 (PMC8387940; doi:10.3389/fmed.2021.733657)

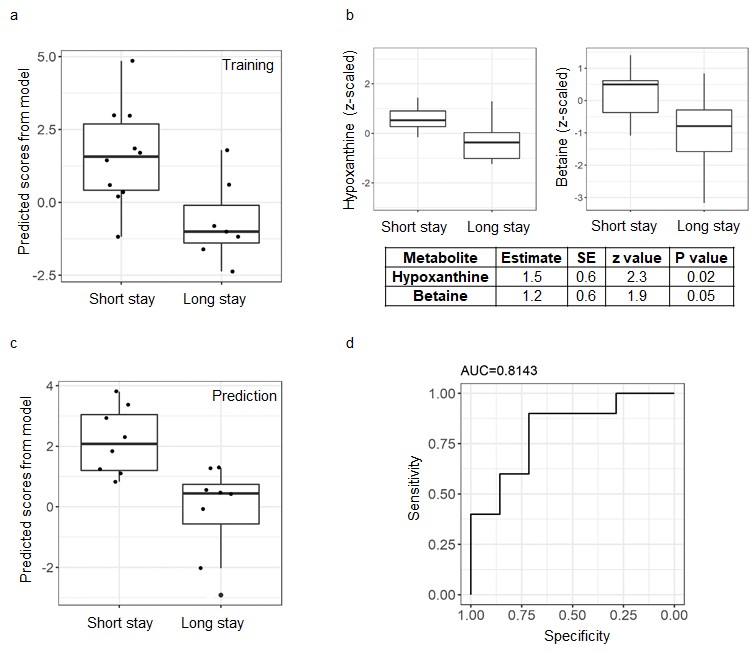

Supplement: Supplementary Figure 1 — A predictive model of length of ICU stay (LOS) based on measurement from day seven. The predictive model trained on the training set (n = 17) (a) featured the same two explanatory metabolites as its counterpart from day one in Figure 2. The model was validated using a prediction set (n = 16) (c), and the AUC value from ROC curve analysis was 0.81 (d). Data points were slightly scattered across the x-axis for ease of visualization in all boxplots. [file Image_1.JPEG]
